# School factors and student drinking in high schools: a cross-sectional study of school policies and party regulation

**DOI:** 10.1186/s12889-020-8317-5

**Published:** 2020-02-17

**Authors:** Veronica S. C. Pisinger, Pernille Bendtsen, Morten Hulvej Rod, Janne S. Tolstrup

**Affiliations:** 1 National Institute of Public Health, University of Southern Denmark, Studiestræde 6, DK-1455 Copenhagen, Denmark; 2 The Council on Health and Disease Prevention, Kristianiagade 12, Copenhagen, Denmark; 3 Steno Diabetes Center Copenhagen, Niels Steensens Vej 2, 2820 Gentofte, Denmark

**Keywords:** Alcohol policies, Alcohol use, Young people, High school, Party regulation, Alcohol price

## Abstract

**Background:**

The effectiveness of school alcohol polices may be affected by the degree of strictness of rules, how they are implemented and enforced, students’ perception of the rules and the consequences of breaking them. The aim of the study was to test the hypothesis that more liberal school alcohol policies, lack of knowledge of the alcohol policy, lower prices of alcohol at school parties, and liberal party regulation were associated with more drinking among high school students.

**Methods:**

Participants were high school students (*n* = 68,898), participating in the Danish National Youth Study in 2014. Data came from questionnaires answered by high school students and school headmasters. Zero-inflated negative binominal regression with clustering of schools (*n* = 117) was used to assess the associations between alcohol policy reported by school headmaster and weekly alcohol intake reported by students. Multilevel negative binominal regression was used to assess the associations between alcohol price and liberal party regulations and units consumed at the last school party and units consumed at the school during the last school party.

**Results:**

In general, school alcohol policies were not associated with high school students’ weekly alcohol intake. High school students who did not know the school alcohol policy had a higher weekly alcohol intake (0.16 drinks 95% CL [0.11;0.21] *p* = 0.000), compared to students who knew the policy. Lower beer prices were positively associated with the number of drinks consumed at the school (*p* = 0.004), but not with the total amount consumed at the last school party (*p* = 0.728). High school students who agreed that students who were drunk could buy alcohol had a higher alcohol intake at the last school party (OR = 0.20 drinks 95% CL [0.18;0.21], *p* < 0.001) and drank more at the school (0.17 drinks 95% CL [0.15;0.18], p < 0.001) compared to those who did not agree that students who were drunk could buy alcohol.

**Conclusion:**

School alcohol policies were generally not associated with drinking among high school students, whereas students’ lack of knowledge of the school policy was associated with a higher weekly alcohol intake. An addition, lower prices and liberal party regulation was associated with higher alcohol intake at school parties.

## Introduction

Excessive alcohol use among young people is an international public health concern and developing effective ways of regulating alcohol use is a high priority. There is increasing recognition that alcohol use among young people is influenced by both individual and contextual factors such as social norms and availability and price of alcohol. At the national level, more comprehensive alcohol control policies that regulate the availability and prices of alcohol have been associated with reduced frequency of alcohol use among young people [1–4]. Furthermore, local regulations and policies, such as those in the school or community, may be predictive of alcohol use among young people.

High school students spend most of their daytime at school and the school is one of the most influential socialization domains in young people’s lives. While the primary objective of a school is to educate, it also constitutes a social context where students interact with teachers and other students and share norms and values that may implicitly or explicitly, confer varying levels of approval toward alcohol use, affecting the behaviour of students [5–7]. Alcohol use among students has been shown to vary between schools, even when differences in the composition of students are taken into account, indicating that school factors influence youth alcohol use [8, 9]. In addition to school-level compositional factors, such as socioeconomic position, degree of urbanisation, and concentration of students with ethnic minority background, which have been found to be associated with heavy drinking [6, 7], students at the same school are also affected by policies and social norms that may predict alcohol use. However, little is known about which school-level policies are effective in preventing excessive alcohol use among students. The effectiveness of school alcohol polices may be affected by the degree of strictness of rules, how they are implemented and enforced and how students perceive the rules and consequences of breaking them [10].

Danish adolescents have one of the highest prevalence of drunkenness among adolescents in Europe [11]. Among Danish high school students 28% (35% of boys and 24% of girls) have been binge drinking frequently (drinking five or more units of alcohol at the same occasion four or more times within the last 30 days) and 20% exceed the Board of health’s’ high risk drinking limits of 21 units a week for men and 14 units a week for women [12]. Alcohol is an integral part of Danish high school culture. At most Danish high schools, alcohol play an integral role in the many social activities that make up the core of Danish high school culture, such as high school parties and study trips [13]. Students are allowed to drink and buy alcohol at high school parties independently of age, because the national age limit for purchasing alcohol in shops (16 years) is not enforced at high school parties [14]. High schools host regular school parties (around five to ten per year) and pre-parties with heavy pre-dinking before going to the school party are common. However, within recent years high schools have adopted alcohol policies in order to reduce and prevent excessive drinking among students.

In this study we use data from multiple data sources to obtain a comprehensive picture of alcohol policies at Danish high schools. We want to analyse the association between alcohol policies and alcohol use among students, as well as the association between students’ knowledge of the policy, alcohol prices at school parties and liberal regulation at school parties, and alcohol intake among students at school parties. We hypothesize that more liberal school alcohol policies, students’ lack of knowledge of the policy, lower prices and liberal regulation at school parties are associated with higher alcohol intake among students.

## Methods

### Study population

#### The Danish National Youth Study 2014- student questionnaire

The data came from the Danish National Youth Study 2014, a national survey of 75,858 high school and vocational school students. The Danish National Youth Study was conducted with the aim of investigating health, health behaviour and mental health among secondary education students in Denmark. All of Denmark’s 137 general high schools and the 12 largest vocational schools were invited to participate. Only high schools were included in this study. The school participant proportion was 87% and the individual participant proportion was 84% in high schools. Teachers gave students a code to access the electronic questionnaire and students answered the questionnaire in class during one to two lessons lasting 45 min each.

#### The Danish National Youth Study 2014- school headmaster questionnaire

In addition to the student questionnaire school headmasters were also invited to complete a questionnaire on school strategies for improving health and well-being, and the rules and general practice of schools when dealing with smoking and alcohol use among students. Of the 119 high school headmasters, 117 answered the school headmaster questionnaire. A thorough description of the study is reported elsewhere [15].

#### Extended alcohol policy study – student mobile questionnaire

In some of the participating schools a more comprehensive examination of the schools’ party regulation and alcohol control policies were conducted. Just over half of the high schools (*n* = 61) were randomly selected to participate in this more detailed alcohol study. In order to obtain data on party regulation and alcohol prices three to five students at each high school were asked to complete a short mobile questionnaire at the party (Friday) and after the party (Monday) (see Additional file 1). This was done in 56 out of the 61 high schools.

Information from the school headmaster questionnaire and student mobile questionnaire were merged on to the student questionnaire based on school. See Table 1 for an overview of data sources used in the study. Participants without school headmaster information (2 schools, *n* = 925) and those with missing data on weekly alcohol intake (*n* = 851) were deleted from the analyses leaving a total study population of 68,898 for further analyses**.**

**Table 1:** Data sources and information on policies, party regulation, prices and alcohol use

	School headmaster questionnaire	Mobile questionnaire on students’ observations	Students questionnaire
N	117	56	68,898
Policies	x		
Students knowledge of the alcohol policy			x
Prices		x	
Party regulation			x
Alcohol use			x

### Measures

#### Alcohol use (student questionnaire)

Alcohol use was characterised by three measures.

*Weekly alcohol intake:* participants were asked how many alcoholic drinks (12 g of pure alcohol) they typically drink on each day of the week. A total weekly alcohol intake variable was calculated summing the daily intake.

*Units consumed during the last school party:* participants who had been to a school party were asked how many alcoholic drinks they consumed at the last school party (including drinking at pre-parties and after-parties) (0, 1–2, 3–5, 6–9, 10–12, 13–15, 16–19, 20 or more). For each category the mid-point value was taken. Students who had never attended a school party were excluded (*n* = 4677), leaving *n* = 63,820 for analysis.

*Units consumed at school during the last school party:* participants were asked how many of these drinks they consumed at the school during the last school party (excluding drinking at pre-parties and after-parties outside the school). Those who did not drink during the last school party and those who had never attended a school party were excluded, leaving *n* = 58,308 for analysis.

#### School-level alcohol policy (school headmaster questionnaire)

School headmasters were asked ‘Has the school adopted an alcohol policy?’ (Yes/No) and whether ‘The alcohol policy is communicated to new students?’, if ‘It is checked if students comply with the alcohol policy’, and ‘Does it have consequences if students do not comply with the alcohol policy?’. The answers were coded ‘Yes’ for “Always/almost always” and “Often”, and ‘Not consistently’ for “Sometimes” and “Never or almost never”. School headmaster reported whether students were allowed to drink at introduction trips, study trips, educational events outside school hours, social events Monday to Thursday, and social events Fridays and Saturdays. Answers were dichotomised into ‘Yes’ (“Yes, always” and “Yes, sometimes”) and ‘No’ (“No” and “Do not arrange”). School headmasters were asked to indicate who was served alcohol at school events with the possible answers “Everyone”, “Everyone older than 16 years”, “everyone older than 18 years”, and “specific classes”.

#### Students’ knowledge of the alcohol policy (student questionnaire)

*Students’ knowledge of the alcohol policy* was measured in the student questionnaire by the question ‘Does your school have an alcohol policy?’ with the possible answers ‘Yes’, ‘No’ and ‘Don’t know’.

#### Prices at parties (student mobile questionnaire)

*Alcohol price* was based on self-reported data on beer and alcopops from students who participated in the school party. At each school, between three and five students were asked to answer a short mobile questionnaire at the party and after the party. The questionnaire included questions about alcohol regulation at school parties, prices in the bar and whether there were adults in the bar. Prices on wine and spirits were excluded from the analyses due to missing data (e.g. few schools sold spirits). Students were asked to take photos of the bar menu and send it to the researchers. The pictures were used to validate students’ answers where there was discrepancy in students’ answers in the questionnaire.

*Bar staff/adults in the bar:* as well as bar prices the students were asked to specify who sold alcohol at the bar.

#### Party regulation (student questionnaire)

*Party regulation* was also measured by two items included in the student questionnaire. Participants were asked how much they agreed with the following: ‘Students are sent home if they are very drunk’, and ‘Students who are drunk can buy alcohol ’. Responses were dichotomized into ‘Agree’ (“Totally agree” and” Agree”) and ‘Do not agree’ (“Neither disagree or agree”,” Disagree”, and” Totally disagree”). “Neither disagree nor agree” was coded conservatively to ‘Disagree’.

### Statistical analyses

We analysed data using multilevel modelling, thereby taking into account the hierarchical data structure. We had three outcomes: 1) weekly alcohol intake 2) units of alcohol consumed during last school party and 3) units consumed at school during the last school party. Zero-inflated negative binominal regression with clustering of schools (*n* = 117) was used to assess the associations between alcohol policy reported by school headmasters and weekly alcohol intake among students, as well as the associations between students’ knowledge of the alcohol policy and students’ weekly alcohol intake. Weekly alcohol intake was non-normally distributed with excessive zeros. The Vuong test showed that a zero inflated negative binominal model was preferred to a standard negative binominal regression model (*p* < 0.001). Multilevel negative binominal regression was used to assess the associations between alcohol price and students’ perceptions of party regulation and units consumed at the last school party and units consumed at the school during last school party. A two-level random intercept model was applied with students (level 1) nested within schools (level 2). All analyses were adjusted for age (continuous) and gender (boys/girls) and performed in STATA 15.

## Results

The study population comprised 68,898 high school students, 61% of whom were girls. Mean age of the students was 17,9 years and 90% considered themselves to be of Danish ethnicity. Most students lived with both parents (64%), 29% lived with one parent and 6% lived alone (Table 2). The mean weekly number of units of alcohol consumed was 13 units among boys and 9 units among girls. The mean number of drinks consumed at last school party was 11 drinks. Two out of three students (68%) who had attended a school party drank more than five drinks at last school party and 24% drank more than five drinks at the school (data not showed).

**Table 2:** Descriptive characteristics of the study population

	Total (%)	Boys (%)	Girls (%)
	68,898 (100)	26,941 (39)	41,957 (61)
Age			
≤ 16 years	14,000 (20)	4912 (18)	9088 (22)
17 years	23,039 (33)	8732 (32)	14,307 (34)
18 years	20,791 (30)	8331 (31)	12,480 (30)
≥ 19 years	11,068 (16)	4986 (19)	6082 (15)
Mean age in years (SD)	17.9 (1.6)	18.0 (2.0)	17.8 (1.3)
Perceived ethnicity ^a^, N (%)			
Danish	61,331 (90)	23,493 (89)	37,838 (91)
Danish and other	4666 (6.9)	1986 (7.5)	2680 (6.5)
Other than Danish	1937 (2.9)	943 (3.6)	994 (2.4)
Living situation^a^, N (%)			
Lives with both parents	44,270 (64)	17,672 (66)	26,598 (64)
Lives with one parent	19,898 (29)	7458 (28)	14,440 (30)
Lives alone	4065 (6.0)	1527 (5.7)	2538 (6.1)

^a^Some columns do not sum to 100% due to missing values

A total of 97% of school headmasters reported that they had adopted an alcohol policy (Table 3). Not adopting an alcohol policy was not associated with weekly alcohol use among students. In addition, 89% reported that the policy was communicated to new students and 87% reported checks were made on students’ compliance with the alcohol policy. Overall, 93% of the school headmasters reported that there were consequences if students failed to apply with the rules. Not communicating or enforcing the policy was not significantly associated with higher weekly alcohol intake among students. All school headmasters reported that alcohol was not allowed on introduction trips, except for two schools that did not arrange introduction trips (data not showed). Most schools allowed students to drink on study trips (85%), however this was not associated with higher weekly alcohol intake among students. Allowing alcohol at educational events after school hours and at social events from Monday to Thursday was also not associated with higher weekly alcohol intake among students. Students in schools where the school headmasters reported that alcohol was always or sometimes served at social events on Fridays or Saturdays (95%) had higher weekly alcohol intake (0.10 drinks 95% CL [0.01;0.18] *p* = 0.025), compared to students in schools where the school headmaster reported that alcohol was not sold at social events at the weekend (5%). Some school headmasters reported that there were restrictions on who could buy alcohol at school events (26%). However, setting restrictions in terms of age limits on who could buy alcohol was not associated with students’ weekly alcohol intake.

**Table 3:** Alcohol policies from school headmaster questionnaire and weekly alcohol intake in students

	School headmasters*	Students	Weekly alcohol intake	
N(%)	117 (100)	68,898 (100)	β (95% CL)	p
Has the school adopted an alcohol policy a specific alcohol/drug policy or part of code of conduct?				
Yes	11 4 (97)	67,654 (98)	Ref.	
No	3 (2.6)	1244 (1.8)	0.08 (−0.05;0.20)	0.233
Is the alcohol policy communicated to new students?				
Not consequently	7 (6.0)	3581 (5.2)	0.02 (−0.08;0.12)	0.706
Yes	110 (89)	65,317 (95)	Ref	
Is it checked that students comply with the alcohol policy				
Not consequently	15 (13)	7785 (11)	−0.00 (−0.04;0.04)	0.888
Yes	102 (87)	61,113 (89)	Ref	
Does it have consequences if students do not comply with the alcohol policy				
Not consequently	8 (6.8)	3756 (5.5)	0.04 (−0.02;0.10)	0.234
Yes	109 (93)	65,142 (95)	Ref	
Are students allowed to drink alcohol…				
on study trips				
No	17 (15)	9456 (14)	Ref.	
Yes	99 (85)	59,155 (86)	−0.00 (− 0.06;0.05)	0.937
…at educational events after school hours				
No	89 (78)	51,191 (77)	Ref.	
Yes	24 (21)	15,593 (23)	0.02 (−0.02;0.06)	0.422
…at social events Monday to Thursday				
No	92 (79)	54,917 (80)	Ref.	
Yes	24 (21)	13,694 (20)	0.03 (−0.03;0.08)	0.324
…at social events Friday or Saturday				
No	6 (5.2)	3190 (4.7)	Ref.	
Yes	110 (95)	65,421 (95)	0.10 (0.01;0.18)	0.025
Who are served alcohol at school events				
Everyone older than 16 years/ 18 years/ specific classes	30 (26)	17,898 (26)	Ref.	
Everyone	85 (74)	50,613 (74)	−0.01 (−0.05;0.03)	0.619

Adjusted for age and gender. *The column does not always sum to 117 due to missing answers on some questions

Most students (64%) answered that their school had an alcohol policy, while 4,3% disagreed and 31% did not know whether their school had an alcohol policy (Table 4). Compared to students’ who knew that their school had an alcohol policy, students who disagreed that their school had an alcohol policy had a higher weekly alcohol intake (0.16 drinks 95% CL [0.11;0.21] *p* = 0.000). Not knowing whether the school had an alcohol policy was not associated with a higher weekly alcohol intake, compared to students who knew their school had an alcohol policy.

**Table 4:** Students’ knowledge of the school alcohol policy

	Students	Weekly alcohol intake	
N(%)	68,731 (100)	β (95% CL)	p
Does your school have an alcohol policy?			
Yes	44,261 (64)	Ref.	
No	2920 (4.3)	0.16 (0.11;0.21)	0.000
Don’t know	21,550 (31)	0.01 (−0.01;0.03)	0.517

Adjusted for age and gender

Lower prices for beer and alcopops were not associated with a higher total alcohol intake at school parties (including units consumed before, during, and after the school event) (Table 5). However, lower prices for beer were positively associated with alcohol intake at the school. For example, students in schools with lower beer prices (10 DKK or less) drank more at the school (0.43 drinks 95% CL [0.18;0.68]; test for trend *p* = 0.004) compared to students in schools with higher beer prices (25 DKK or more). The same tendency with higher alcohol intake at school were also seen with higher alcopop prices, however the test for trend was not significant (*p* = 0.071). Having adults in the bar or offering more types of alcoholic drinks were not associated with units consumed at last school party (data not shown).

**Table 5:** Alcohol prices at school parties and units consumed at last school party and units consumed at school at last school party

	Schools	Students	Total units consumed at last school party		Units consumed at the school during last school party	
			Coefficient, β (95% CL)	p	Coefficient, β (95% CL)	p
N (%)	56 (100)	35,378 (100)	32,850		30,142	
Beer (Danish Krone, DKK)						
10 or less	6 (11)	2160 (6.1)	−0.03 (− 0.15;0.08)	0.728	0.43 (0.18;0.68)	0.004
15	12 (21)	8201 (23)	−0.01 (− 0.10;0.08)		0.11 (− 0.09;0.31)	
20	25 (45)	16,526 (47)	−0.04 (− 0.12;0.04)		0.11 (− 0.06;0.30)	
25 or more	13 (23)	8491 (24)	Ref.		Ref.	
Alcopops (DKK)						
20 or less	11 (22)	5710 (18)	−0.09 (−0.18;0.00)	0.071	0.16 (−0.02;0.33)	0.071
25	21 (42)	14,501 (46)	−0.01 (− 0.08;0.06)		0.11 (− 0.04;0.26)	
30 or more	18 (36)	11,306 (36)	Ref.		Ref.	

Adjusted for age and gender

Students who did not agree that students are sent home if they are very drunk (50%) had a higher alcohol intake at the last school party (0.05 drinks 95% CL [0.04;0.06], *p* < 0.001) compared to those who agreed that students are sent home if they were very drunk (Table 6). However, students who did not agree that students are sent home if they are very drunk did not drink more at the school. Students who agreed that students who are drunk can buy alcohol (64%) had a higher alcohol intake at the last school party (OR = 0.20 drinks 95% CL [0.18;0.21], *p* < 0.001) and drank more at the school (0.17 drinks 95% CL [0.15;0.18], *p* < 0.001) compared to those who did not agree that students who are drunk can buy alcohol.

**Table 6:** Students’ perceived party regulation at school parties and units consumed at last school party and units consumed at school during last school party

	Students	Total units consumed at last school party		Units consumed at school during last school party	
		Coefficient, (95% CI)	p	Coefficient, (95% CI)	p
N(%)	63,868 (100)	63,712		58,216	
Students are sent home if they are very drunk					
Do not agree	32,016 (50)	0.05 (0.04;0.06)	0.000	−0.01 (−0.02;0.01)	0.443
Agree	31,852 (50)	Ref.		Ref.	
Students who are drunk can buy alcohol when					
Do not agree	23,220 (36)	Ref.		Ref.	
Agree	40,625 (64)	0.20 (0.18;0.21)	0.000	0.17 (0.15;0.18)	0.000

Adjusted for age and gender

## Discussion

In this study of 68,898 high school students in 117 Danish high schools we found that in general school alcohol policy was not associated with students drinking, whereas students’ lack of knowledge of school alcohol policy, lower prices for beer at school parties and liberal party regulation was associated with more drinking among students.

### Allowing alcohol to be sold at social and educational events at the school

In Denmark, students can buy and drink alcohol at high school parties independent of age as the general age limit for purchasing alcohol (16 years) does not apply for private events. Most high schools allow alcohol at social events during the weekend (95%) and generally do not have strict rules on alcohol use. Allowing alcohol at social events at the school on Fridays and Saturdays was associated with higher weekly alcohol intake among students, compared to schools that did not allow alcohol at social events during the weekend. In line with this result, research among slightly older young people in the US showed that students attending colleges that ban alcohol were less likely to binge drink and more likely to abstain from alcohol [16–18]. However, allowing alcohol at social events between Monday and Thursday and at educational events after school hours was not associated with higher alcohol intake. In line with these results, a Dutch study detected no differences in rates of heavy episodic binge drinking among secondary school students attending schools with a total ban on drinking compared with schools that allowed student drinking on certain occasions [19]. In addition, as this is a cross sectional study the lower weekly alcohol intake among students at schools that did not allow alcohol at social events during the weekend might be due to self-selection of low consumption students to schools that do not allow alcohol at social events. The 5% of schools that did not allow alcohol at social events during the weekend did not have a different composition of students with regard to age, sex or ethnicity, but did have a higher proportion of students who identified as Christians.

### Prices on alcohol at school parties

In line with our findings, the price of alcohol has previously been found to be an important factor in drinking among college students. Low prices and easy access to alcohol has been found to be strongly correlated to binge drinking among college students [16]. Lower beer prices were significantly associated with higher alcohol intake at the school, whereas lower alcopop prices were not significantly associated with higher alcohol intake at the school. This could be due either to generally higher prices for alcopops compared to beer, or lower statistical power as fewer schools served alcopops compared to beer. Lower beer prices at school parties were associated with more drinking at the school, however the total consumption of beer at the last school party was not higher. Pre-parties with heavy pre-dinking are widespread among Danish high school students. The result therefore suggests that students at schools with higher alcohol prices at school parties preload to a higher extend than students at schools were alcohol prices at the school party are lower. Alcohol prices in Denmark are generally low and so is the legal purchasing age of 16 years. Alcohol is also easily accessible with high outlet density and low control of legal purchasing age. Danish high school students therefore have easy access to cheap alcohol outside school, which makes it difficult for high schools to regulate students’ alcohol intake at school parties by charging higher alcohol prices at the school. Having an age limit on who can by alcohol at the school party and having adults in the bar were also not associated with lower alcohol intake among students. This could support the notion that the largest proportion of drinking occur before or after the actual party at the school, thereby making preventive actions at the school party less likely to affect students’ drinking.

### Perception of policy enforcement and availability of alcohol at school parties

Liberal party regulation was associated with a higher alcohol intake at school parties. Students who disagreed that students are sent home if they are very drunk were associated with higher total alcohol intake at the last school party, but not higher alcohol intake at the school. Students who agreed that students who are drunk can buy alcohol had a higher alcohol intake at the last school party, both in total and at the school. The result could possibly be due to reversed causation as only students who drink more at parties know whether they are sent home or can buy alcohol when they are drunk. This could also indicate that students’ perception of enforcement of the alcohol policy and the accessibility of alcohol might be crucial and may be more important than formal rules. University of Washington professor of Social Work Richard Catalano and colleagues [10] studied whether anti-alcohol policies in public and private schools in the state of Washington and in the state of Victoria in Australia were effective for eighth and ninth graders. They found that each school’s individual policy mattered less than the students’ perceived enforcement of it. Thus, even if a school had a suspension or expulsion policy, if students felt that the school did not enforce it then they were more likely to drink on campus. But, even if a school’s policy was less harsh – such as requiring counselling – students were less likely to drink at school if they believed that school officials would enforce it [10]. Their results are supported by Harris et al. [20] who also found that enforcement of a newly introduced alcohol policy in colleges was associated with a decline in heavy episodic drinking among students in their longitudinal study. In contrast to previous studies that have found adopting a school alcohol control policy to be associated with less drinking among students [9, 21], we did not find any association between control of the policy and consequences if the policy was violated reported by school headmaster and students’ weekly alcohol intake. However, we found that students’ knowledge of the alcohol policy appears to be associated with the level of drinking among students. Students who answered that their school did not have an alcohol policy had a higher weekly alcohol intake as compared to students who agreed that their school had an alcohol policy. This could support the finding that students’ knowledge of the policy and perception of enforcement are very important factors for the effectiveness of school alcohol policies on students’ alcohol use.

### Study strengths and limitations

One of the major strength of this study is that data came from several different sources. By including data on school policy from school headmasters, student observation at school parties and questionnaire data on drinking among students, we were able to give a comprehensive and detailed picture of how different school policies affect students’ drinking. In addition, this study included a large number of schools (*n* = 117) and students (*n* = 68,898), which reduced the risk of random errors.

The study also had limitations that need to be noted. The data was cross-sectional and so it is not possible to make causal inferences from the noted associations. We hypothesized that liberal alcohol policies would reflect social norms at the school and be associated with a higher level of drinking among students, however schools with students who drink excessively might introduce more restrictive policies to reduce students drinking and our results could therefore be affected by reverse causation. This is an important limitation of the design and prevents strong inference of the results. The use of self-reported data for student drinking and alcohol policies is a potential source of bias. School headmasters may report stricter alcohol policies and students may report lower alcohol intake as a result of socially desirable responding. Studies among adults have shown that self-reports of alcohol consumption are generally underestimated [22], and therefore students drinking might have been underrated. School headmasters may also report stricter alcohol policies due to a socially desirable wish to portray themselves as a responsible headmasters. Misclassification of school policies and students drinking could potentially have blurred the associations between school alcohol policies and students drinking. In addition, the validity of the observations from the mobile questionnaire conducted among students at school parties could have been compromised by the fact that most students had been drinking at school parties. The validity of students’ reports was pilot tested by comparing students’ answers to researchers’ answers after a school party where two researchers and four students participated and completed the questionnaire. The level of accordance was generally high, but in order to increase validity students were asked to send photos of the bar menu to the researchers to validate their answers.

## Conclusion and implications

In general school alcohol policy was not associated with drinking among students, though schools that allow alcohol at social events during the weekend have a higher weekly alcohol intake among students compared to those that do not allow alcohol at social events during weekends. A lack of knowledge of the alcohol policy among students and liberal party regulation were associated with more drinking among students. Our results indicate that students’ knowledge of the policy and perception of enforcement are important factors for the effectiveness of school alcohol policies on students’ alcohol use.

Lower beer prices at school parties were associated with more drinking at the school, however the total consumption of beer during the night of the school party was not higher. The result therefore suggests that students at schools with higher alcohol prices at school compensate with a higher alcohol intake before or after the school party than students at schools were alcohol prices at the school party are lower. The easy access to cheap alcohol outside school makes it difficult for high schools to regulate students’ alcohol intake at school parties by charging higher alcohol prices at the school. This suggests that national regulations governing alcohol purchasing ages and prices need to support the effort of high schools to reduce excessive drinking among high school students.

More research into effective school-level policies is needed in order to prevent and reduce excessive alcohol use among students.

## Supplementary information

**Additional file 1.** Alcohol policies at high schools (to be filled in by students). Party observation questionnaire.

## Data Availability

The datasets generated and analyzed during the current study are not publicly available due to sensitivity. of the data but are available from the corresponding author on reasonable request.

## Abbreviation

DKK: Danish Krone
