# Supplementary material for: School factors and student drinking in high schools: a cross-sectional study of school policies and party regulation
Source: BMC Public Health. 2020 Feb 17;20:236. doi: 10.1186/s12889-020-8317-5 (PMC7027101; doi:10.1186/s12889-020-8317-5)
Supplement: Supplementary file 1 — Additional file 1. Alcohol policies at high schools (to be filled in by students). Party observation questionnaire. [file 12889_2020_8317_MOESM1_ESM.docx]

**Alcohol policies at high schools (to be filled in by students)**

**PARTY OBSERVATION QUESTIONNAIRE**

**1. Name and mobile number:**

_______________________________________________

**2. School name:**

_______________________________________________

**3. Date of school party:**

_______________________________________________

**4. Is there a specific theme for the party?**

| Yes | **□** |
| --- | --- |
| No | **□** |

If tes, what is the theme _______________________________________________

**5. What is the price on: Is not for sale**

Bottled beer _____________ **□**

Tap beer (1/4 liter) _____________ **□**

Tap beer (1/2 liter) _____________ **□**

A shot _____________ **□**

A drink _____________ **□**

A glas of wine _____________ **□**

Sommersby, breezer, lign. _____________ **□**

Soda _____________ **□**

Water _____________ **□**

Other _____________

***Please take a picture of the bar menu and send it to the research team.***

**6. Can you by discount card/vouchers for beer og drinks?**

| Yes | **□** |
| --- | --- |
| No | **□** |

If yes, what kind of discount? (please write price and number of drinks)

_______________________________________________

**7. Who is in the bar?**

Students **□**

Teachers **□**

Others ________________________

***Please take a picture of the bar and send it to the research team.***

**8. Is it possible to bring your own alcohol to the party?**

| Yes | **□** |
| --- | --- |
| No | **□** |
| Dont know | **□** |

_____________________________

**9. Do you think anybody brought their own alcohol?**

| Yes | **□** |
| --- | --- |
| No | **□** |
| Dont know | **□** |

_____________________________

**10. Did you see anybody smoking cannabis at the party?**

| Yes | **□** |
| --- | --- |
| No | **□** |
| Dont know | **□** |

_____________________________

**11. Did you see anybody take other drugs at the party?**

| Yes | **□** |
| --- | --- |
| No | **□** |
| Dont know | **□** |

_____________________________

**12. Can you buy food at the party?**

| Yes | **□** |
| --- | --- |
| No | **□** |
| Dont know | **□** |

_____________________________

***The next questions conserns the teachers at the party***

**13. Is one or more teachers present at the party?**

| Yes | **□** |
| --- | --- |
| No | **□** |

If yes, how many?_______________________________________________

***If there are teachers present at the party, please answer the next question otherwise jump to question 15.***

**14. To what extent do you agree with the following statements**:

|  | Strongly agree | Agree | Neither agree nor disagree | Disagrre | Strongly disagree |
| --- | --- | --- | --- | --- | --- |
| The teachers keep an eye on us | **□** | **□** | **□** | **□** | **□** |
| The teachers make sure we do not drink too much | **□** | **□** | **□** | **□** | **□** |
| The teahcers drink alcohol themselves at the party | **□** | **□** | **□** | **□** | **□** |
| Some teachers smoke at the party | **□** | **□** | **□** | **□** | **□** |
| The teachers party with us | **□** | **□** | **□** | **□** | **□** |

**15. Try to discribe the party atmosphere and process with your own words** (What time do people arrive, is it packed with people, how is the bar, dj, band etc)

**Please write here if you have further comments**

Thank you!

**MONDAY QUESTIONNAIRE**

**1. Name and mobile number:**

_______________________________________________

**2. School name:**

_______________________________________________

**3. Date of school party:**

_______________________________________________

**4. How many people participated at the party last freday? (if you dont know ask the members of the party committe)?**

Less than 100 **□**

100-200 **□**

200-300 **□**

300-400 **□**

400-500 **□**

500-600 **□**

600-700 **□**

More than 700 **□**

***The next questions concern the school parties at your school in general***

**5. Do you have professional guards?**

| Yes | **□** |
| --- | --- |
| No | **□** |

If yes, what is there primary task of the guards? _______________________________________________

**6. How many parties do your school host a year (check lectio)?**

1 □
2-3 □
4-5 □
6-7 □
8-9 □
10 or more □
Don’t know □

**7. Is it possible to buy alcohol at all your parties at school?**

| Yes | **□** |
| --- | --- |
| No | **□** |
| Don’t know | **□** |

_____________________________

**8. Do you have other events at your school where it is possible to buy alcohol?**

| Yes | **□** |
| --- | --- |
| No | **□** |
| Don’t know | **□** |

If yes what kind of events (e.g. band nights, cafes or other)? _______________________________________________

**9. Do you have alcohol free events at your school?**

| Yes | **□** |
| --- | --- |
| No | **□** |
| Don’t know | **□** |

If yes what kind of events (e.g. band nights, cafes, talks or other)? _______________________________________________

**10. Can you smoke at school parties**

| Yes | **□** |
| --- | --- |
| No | **□** |

**11. Where are you allowed to smoke at school parties?**Inside at the party **□**

In special smoking areas/rooms inside **□**

Outside in special smoking areas at the school **□**

Outside (everywhere) **□**

Outside not at the school **□**

**12. Do people smoke at school parties?**

Yes, inside at the party **□**

Yes, in special smoking areas/rooms inside **□**

Yes, at the toilets **□**

Yes, outside in special smoking areas at the school **□**

Yes, outside (everywhere) **□**

Yes, outside not at the school **□**

No, no one smokes at parties at the school **□**

**1. What happens if people get caught smoking at school parties?**

**_________________________________________________________________________________________________________________________________________________________________________________________________________________________________**

**14. What time do the party officially end?**

Kl. ___ :

**15. What time do the party actually end ?**

Kl. ___ :

**16. To what extent do you agree with the following statements**:

|  | Strongly agree | Agree | Neither agree nor disagree | Disagrre | Strongly disagree |
| --- | --- | --- | --- | --- | --- |
| Students who are drunk can buy alcohol | **□** | **□** | **□** | **□** | **□** |
| Students are sent home if they are very drunk | **□** | **□** | **□** | **□** | **□** |
| Adults intervene if groups of students make a fuss for the party | **□** | **□** | **□** | **□** | **□** |
| Adults intervene if a student is drunk very drunk | **□** | **□** | **□** | **□** | **□** |
| It is easy to reenter if you have left the party | **□** | **□** | **□** | **□** | **□** |
| You are not allowed entrance if you are very drunk | **□** | **□** | **□** | **□** | **□** |

**Please write here if you have further comments**

Thank you!
